# Supplementary material for: Amplification Refractory Mutation System (ARMS)-PCR for Waxy Sorghum Authentication with Single-Nucleotide Resolution
Source: Foods. 2021 Sep 18;10(9):2218. doi: 10.3390/foods10092218 (PMC8467681; doi:10.3390/foods10092218)
Supplement: Supplementary file 1 [file foods-10-02218-s001.zip › foods-1353494-supplementary.pdf]

Supporting information

Amplification refractory mutation system (ARMS)-PCR for waxy sorghum authentication with single-nucleotide resolution to avoid contaminants

Xiaoying Zhu[1], Minghua Wu[1], Rujie Deng[1,*], Mohammad Rizwan Khan[3],Sha Deng[1], Xi Wang[2,*], Rosa Busquets[4],Wanyu Deng[1], Aimin Luo[1,*]

[1] College of Biomass Science and Engineering, Healthy Food Evaluation Research Center and Key Laboratory of Food Science and Technology of Ministry of Education of Sichuan Province, Sichuan University, Chengdu 610065, China

[2] Sichuan Langjiu Group Co., Ltd., Luzhou 646523, China

[3] Department of Chemistry, College of Science, King Saud University, Riyadh, 11451, Saudi Arabia

[4] School of Life Sciences, Pharmacy and Chemistry, Kingston University London, Penrhyn Road, Kingston Upon Thames KT1 2EE, United Kingdom

*Corresponding author

drj17@scu.edu.cn, wangxi15283088230@163.com, luoam@scu.edu.cn

Table S1. Oligonucleotide sequences

Name	Sequences (5' to 3')
Wxt F0	CAAATACGACGTGTCAACGG
Wxt F1	CAAATACGACGTGTCAACTG
Wxt F2	CAAATACGACGTGTCAAAGG
Wxt F3	CAAATACGACGTGTCAACAG
Wxt F4	CAAATACGACGTGTCACCGG
Wxc F0	CAAATACGACGTGTCAACGT
Wxc F1	CAAATACGACGTGTCAACTT
Wxc F2	CAAATACGACGTGTCAACCT
Wxc F3	CAAATACGACGTGTCAAAGT
Wxc F4	CAAATACGACGTGTCAACAT
R1	TTGTTGCACTCAGAACCAGC

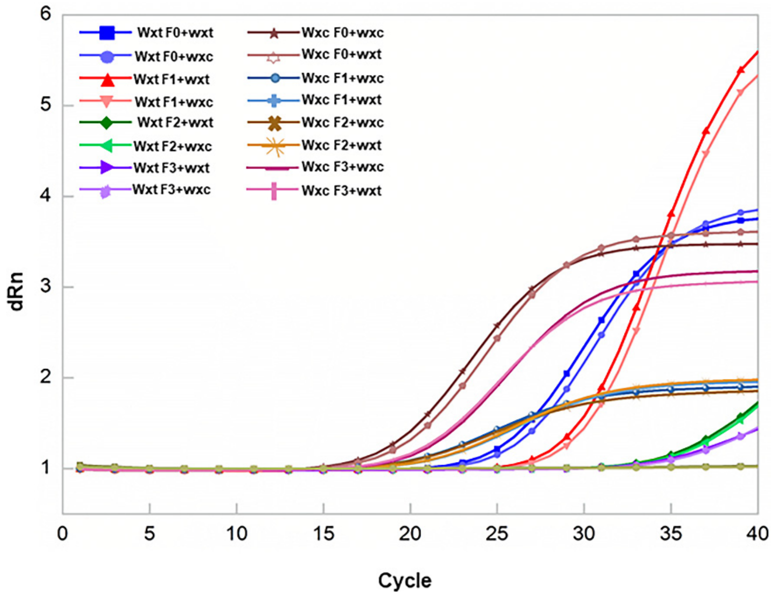

Figure S1. Primers screening. Several allele-specific forward primers designed for wx^c waxy sorghum and non-wx^c sorghum were used for qPCR amplification.

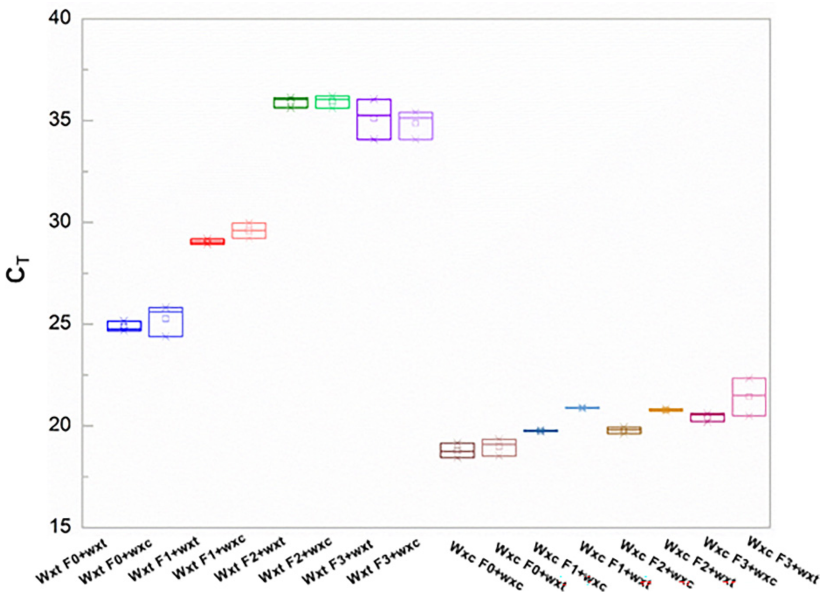

Figure S2. C_T values of qPCR amplification for screening primers that specifically identified wx^c waxy sorghum and non-wx^c sorghum. The allele-specific forward primers wxc F0,F1,F2,F3 and wxt F0, F1, F2 and F3 designed for wx^c waxy sorghum and non-wx^c sorghum could not specifically identify wx^c waxy sorghum or non-wx^c sorghum.

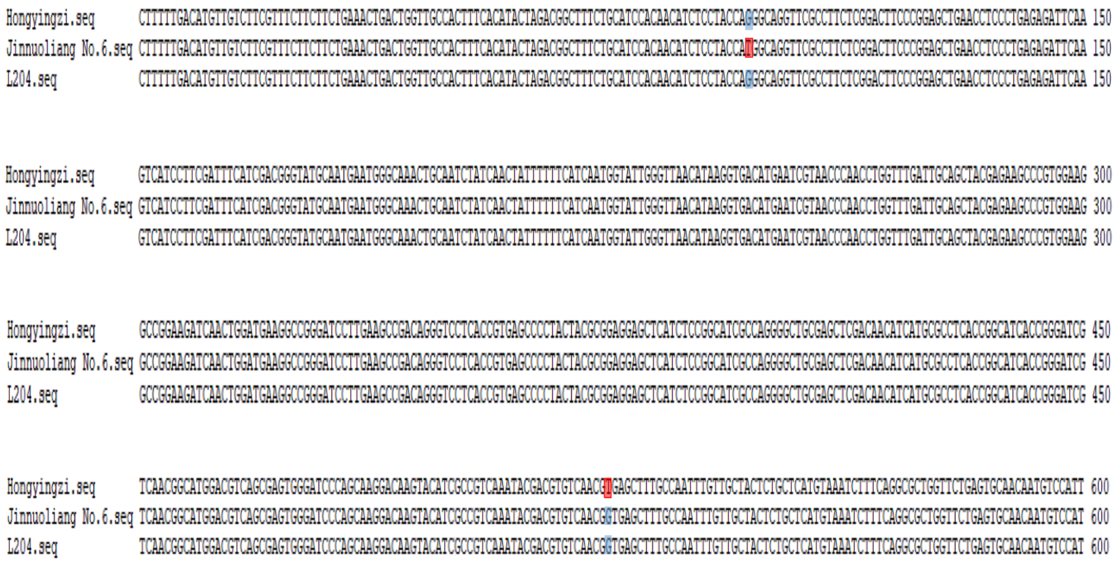

Figure S3. Partial DNA sequence information about *'Hongyingzi'*, *'Jinnuoliang No.6'* and *'Jin 204'*. 'Hongyingzi' was wx^c, and 'Jinnuoliang No.6' was wx^b, and the 'Jin 204' was wild.

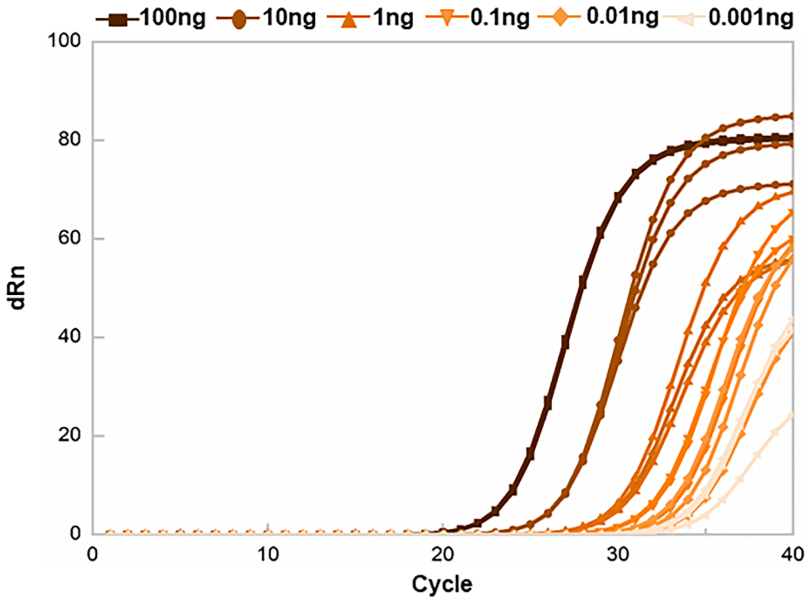

Figure S4. The amplification curves of *'Hongyingzi'* DNA samples that were continuously ten-fold diluted from 100.0 ng to 1.0 pg.

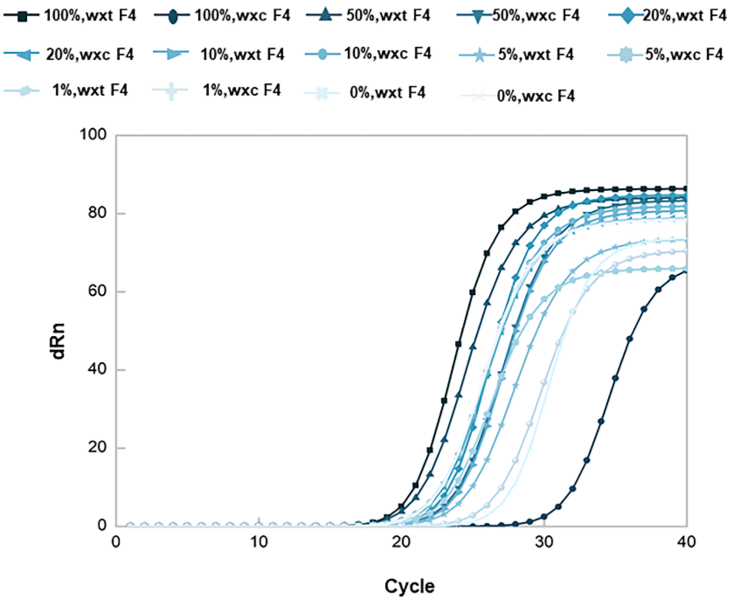

Figure S5. Amplification curves of *'Jinnuoliang No.6'* with different content ratios in mixtures.
